# Risk Factors of Coexisting Septic Spondylitis and Arthritis: A Case-Control Study in a Tertiary Referral Hospital

**DOI:** 10.3390/jcm10225345

**Published:** 2021-11-16

**Authors:** Sheng-Fen Wang, Po-Liang Lai, Hsiang-Fu Liu, Tsung-Ting Tsai, Yu-Chih Lin, Yun-Da Li, Ping-Yeh Chiu, Ming-Kai Hsieh, Fu-Cheng Kao

**Affiliations:** 1 Department of Anesthesiology, Chang Gung Memorial Hospital, College of Medicine, Chang Gung University, Taoyuan 333, Taiwan; wangfenn@gmail.com; 2 Department of Orthopaedic Surgery, Spine Section, Bone and Joint Research Center, Chang Gung Memorial Hospital, Chang Gung University College of Medicine, Taoyuan 333, Taiwan; polianglai@gmail.com (P.-L.L.); hsiangfu.liu@gmail.com (H.-F.L.); tsai1129@gmail.com (T.-T.T.); b101092127@tmu.edu.tw (Y.-C.L.); yunda1222@hotmail.com (Y.-D.L.); jobphage@cgmh.org.tw (P.-Y.C.); mk660628@gmail.com (M.-K.H.)

**Keywords:** infectious spondylitis, septic arthritis, hypoalbuminemia, psoas abscess

## Abstract

Introduction: In patients under immunosuppression or severe sepsis, it is sometimes manifested as coexisting septic arthritis and spondylitis. The aim of this study is to evaluate and investigate the risk factors of infectious spondylitis associated with septic arthritis. Methods: The study retrospectively reviewed the patients diagnosed with infectious spondylitis between January 2010 and September 2018 for risk factors of coexisting major joint septic arthritis. Results: A total of 10 patients with infectious spondylitis and coexisting septic arthritis comprised the study group. Fifty matched patients with solely infectious spondylitis were selected as the control group. Major risk factors include preoperative C-reactive protein (*p* = 0.001), hypoalbuminemia (*p* = 0.011), history of total joint replacement (*p* < 0.001), duration of preoperative antibiotics treatment (*p* = 0.038) and psoas muscle abscess (*p* < 0.001). Conclusion: Infectious spondylitis and septic arthritis are thought of as medical emergencies due to their high mortality and morbidity. Our study evaluated 5 risk factors as significant major findings: hypoalbuminemia (<3.4 g/dL), higher preoperative CRP (>130 mg/L), psoas muscle abscess, longer preoperative antibiotics treatment (>8 days) and history of total joint replacement. Clinicians should pay attention to the patients with those five factors to detect the coexisting infections as early as possible.

## 1. Introduction

Infectious spondylitis is a disease whereby most of the cases arise from hematogenous seeding of the axial skeleton from remote infection foci, such as urinary tract infections [1]. Arterial and venous routes are two of the most common pathways of hematogenous spread [2]. Since the blood vessels to the disc in adults are obliterated, it forms a large avascular structure in the human body. The organisms invade the end-arterial arcades which have relatively slow blood flow adjacent to the disc and then spread into the disc, leading to bone infarction and avascular necrosis in the plate and vertebra [3]. It may further extend from the vertebral body to the paravertebral area, epidural space and continuous vertebral body [4]. A spondylodiscitis is when an infection affects the disc place and spreads to two adjacent vertebral bodies. Occasionally, infection spreading into paraspinal soft tissue would induce secondary epidural abscess, paravertebral abscess, psoas muscle abscess or prevertebral collection [5,6,7]. Further spread might go through the connective tissue or muscle and hematogenous route to other joints such as hip, knee or shoulder and cause septic arthritis.

Septic arthritis is also usually caused by bacteremia [8]. Since the synovial membrane is a vascularized tissue but has a lack of a protective basement membrane, it is vulnerable to the invasion of bacteria [9]. Bacteria may come from injection, trauma, the gastrointestinal system or the genitourinary tract. Organisms invading the human body by the pathway are then infected via hematogenous inoculation through the transphyseal vessels, and then spread into the metaphyseal region and are secondarily infected as the metaphysis is within the joint [10]. The most common cause of septic arthritis, which can easily bind to the cell surface by producing several kinds of adhesins, is *Staphylococcus aureus*, followed by streptococci and other Gram-positive germs [11,12,13]. These bacterial infections cause joint damage since they induce host inflammation and tissue ischemia. The toxin made by these organisms and the reactive oxygen species made by neutrophils also lead to cartilage and tissue damage and autodigestion [14]. The infection and inflammation process eventually leads to purulent accumulation, joint pressure increase and synovial blood flow decrease, resulting in cartilage anoxia.

Septic arthritis and infectious spondylitis are unusual diseases with occasionally uneven symptoms and signs. Due to the high mortality and morbidity, both are considered as important medical emergencies. Irreversible joint destruction and neurological deficits cause devastating and poor prognosis in the event of delayed or inadequate treatment. Early detection and awareness as well as effective treatment are the key to prevent terrible outcomes. However, the incidence of septic arthritis and infectious spondylitis has seemed to increase, even with the advances in imaging techniques. The use of immunosuppressants, increasingly serious antibiotics resistance and growing numbers of specific vulnerable populations, such as the elderly, organ or bone marrow transplant recipients and those with chronic diseases including lupus, rheumatoid arthritis and diabetes mellitus, may all be partly responsible.

In our clinical practice, there are some patients under immunosuppression or severe septic conditions. In such cases, it is sometimes manifested as infectious spondylitis associated with septic arthritis. However, after reviewing many studies and articles, we did not find a study focused on this condition. Therefore, we performed this study to analyze and focus on the risk factors and clinical conditions of coexisting infections of infectious spondylitis and septic arthritis.

## 2. Method

### 2.1. Study Population

The retrospective database review was conducted to identify the patients who had received the surgeries for infectious spondylitis between January 2010 and September 2018. All the procedures were performed in the spine section of the orthopedic department in Chang Gung Memorial Hospital. Inclusion criteria was primary spinal operations for infectious spondylitis debridement. Patients were excluded from secondary infectious spondylitis due to previous spinal surgeries for spine trauma, tumor and other degenerative lesions. The diagnosis of infectious spondylitis was based on the patients’ clinical symptoms, laboratory data, plain films and magnetic resonance imaging (MRI) of the spine.

During the study period, the enrolled patients had to have a minimum follow-up period of one year and were divided into solely infectious spondylitis and coexisting infections groups. Patients with coexisting infections made up the study group, which was infectious spondylitis associated with septic arthritis within 3 months of infection treatment. The septic arthritis of major joints was diagnosed by physical examination accompanied with culture data, laboratory data and imaging studies, including X-ray, computed tomography, magnetic resonance imaging or bone scan. A computer-generated list of potential controls was obtained from the solely infectious spondylitis patients. A pairwise, retrospective, case-control study with one-to-five matching was performed between the study and control groups. Controls were chosen on the basis of the following matching criteria: similar gender, age and the infection and surgical levels. The list of potential controls was reviewed for the best possible match, giving priority to gender, age and the infection and surgical levels. When more than five possible controls existed to match one study case, the five controls with the closest date of admission to that of the study patient were selected.

### 2.2. Data Collection

Medical records, imaging reports, laboratory data, culture reports and operation records were reviewed and analyzed. Magnetic resonance imaging of the spine, including coronary view, sagittal view and axial view, was obtained preoperatively. X-ray imaging of septic arthritis was obtained after clinical symptoms and physical examination revealed suspicion. All procedures performed in studies involving human participants were in accordance with the ethical standards of the institutional and/or national research committee and with the 1964 Helsinki declaration and its later amendments or comparable ethical standards. The Chang Gung Medical Foundation IRB approved this study and waived the requirement for informed consent due to the retrospective nature of the study.

### 2.3. Stastistical Analysis

A statistical software program (SPSS for Windows, version 12.0; SPSS Inc.) was used to analyze the preoperative, operative and postoperative data in both groups. All data were presented as mean ± standard deviations. The differences between groups were assessed using the Mann–Whitney U test for continuous variables and Fisher’s exact test for categorical variables. Statistical significance for all tests was set at a *p*-value of 0.05. Receiver operating characteristic (ROC) curve and cutoff values were calculated for significant factors, with the area under the curve (AUC) proximal to 0.8.

## 3. Result

Between January 2010 and September 2018, a total of 351 patients with infectious spondylitis who underwent a spinal debridement operation were enrolled in this study. Seventy-one patients were excluded because their infectious spondylitis resulted from post-operational, tumor-related or post-trauma infection. From the remaining 280 patients, there were 10 patients diagnosed with infectious spondylitis plus major joint infection (3 septic shoulders, 2 septic hips and 7 septic knees, 2 of them with two major joint infections), referred to as the study group. The mean age of the study group was 63.06 ± 15.03 years. The remainder of the 270 patients were further evaluated and used as the database for the selection of the controls. A pairwise one-to-five matching was performed between the study and control groups. From the 270 patients, 50 patients with complete laboratory and culture data were selected as the control group, according to the following matching criteria with the study group: similar gender, age, the infection level and the surgical level

### 3.1. Pre-Operative Conditions and Laboratory Data

Compared with the control group, the study group revealed significant hypoalbuminemia (*p* = 0.011) and higher preoperative C-reactive protein (CRP) (*p* = 0.001) and platelet (*p* = 0.048) levels. There was no significant difference in comorbidities between the two groups, but patients in the study group had more history of total joint replacement (*p* < 0.001) and longer duration of preoperative antibiotics use (*p* = 0.038). The receiver operating characteristic (ROC) curve and cutoff values were calculated for preoperative albumin, CRP and days of antibiotics use. Then, we found that CRP > 130 mg/L, albumin < 3.4 g/dL and preoperative antibiotics days > 8 predisposed the coexisting infections of infectious spondylitis and septic arthritis. The analysis of preoperative conditions and laboratory data between two groups is shown in Table 1.

### 3.2. Operation Methods and Post-Operative Laboratory Data

There was no significant difference in operative methods between the study and control groups. The posterior approach was the major surgical method of all patients (48/60), and only a few patients needed anterior plus posterior operations for infectious spondylitis (4/60). Moreover, the laboratory data at one month post-operation were investigated and none of them revealed a significant difference. The results of the analysis of operative methods and postoperative laboratory data are listed in Table 2.

### 3.3. Preoperative MRI and Final Culture Results

From the preoperative spine MRI, there was no significant difference in multiple-level infection and infection level (cervical, thoracic or lumbar) between the study and control groups. Only preoperative psoas muscle abscess had a significant effect on coexisting infectious spondylitis and septic arthritis (*p* < 0.001). The summary of the preoperative spine MRI is presented in Table 3. In the analysis of culture results, the major microorganism was *Staphylococcus aureus*, in 26.7% of all patients (16/60), followed by *Streptococcus species*, in 15%. The culture rate was 72% in the control group and 80% in the study group. There was no significant difference in culture rate, *Staphylococcus aureus* and *Streptococcus species* infection rate between the two groups (Table 4).

## 4. Discussion

Coexisting infections in the spine and major joints are associated with a deteriorated infection status. The condition of infectious spondylodiscitis complicated with septic arthritis is rare but usually devastating to patients and clinical physicians. When the infection progresses, microorganisms mainly spread through two routes: the hematogenous route and contiguous spreading. Hematogenous seeding is relatively associated with infectious status, including bacteremia, sepsis status, infection control and individual immune response, while contiguous spreading means that the infection spreads from adjacent tissues into the surrounding structures. According to the results of our study, patients with hypoalbuminemia (<3.4 g/dL), higher preoperative CRP (>130 mg/L), psoas muscle abscess, longer preoperative antibiotics treatment (>8 days) and history of total joint replacement are predisposed to combined infectious spondylitis and septic arthritis. Hypoalbuminemia is a useful way to evaluate poor nutrition status and poor immune response [15,16]. A high level of CRP indicates that the patient is under a severe systemic inflammatory condition, which might present poor infection control, especially after long-term antibiotics treatment [17,18]. In artificial prosthesis, a bio-conditioning film forms shortly after insertion due to host proteins such as fibrinogen, fibronectin and vitronectin absorbed onto the surfaces of orthopedic implants. This state of the biomaterial surface enhances bacterial colonization from hematogenous spreading through interactions between bacterial proteins and host proteins [19]. Infectious spondylitis is indicated as one of the most frequent causes of secondary psoas muscle abscess. Many studies have reported that bacteria spread distally through the psoas muscle as an abscess and then penetrate the hip joint, causing septic arthritis [20].

In the general population, the normal serum concentration of albumin in healthy adults is approximately 3.5 to 5.0 g/dL. Serum albumin is not only a known marker of nutritional status, but it is also recognized as an inflammatory marker [21]. The mechanism of hypoalbuminemia could be caused by a reduction of hepatic synthesis and increased leakage into the interstitial space during the deteriorated infection status [22]. Hypoalbuminemia reflects the physiological stress from disease-related inflammation that commonly occurs in chronic inflammatory conditions and may develop quickly even in well-nourished patients after trauma or acute illness [23]. Albumin enhances antimicrobial defense and injury repair mechanisms with its antioxidative properties, and could facilitate resolution of disease by the formation of anti-inflammatory lipoxins, resolvins and protectins [24]. Many studies have described hypoalbuminemia as a risk factor for poor clinical outcomes in acute illness [25,26]. In the analysis of our results, hypoalbuminemia was significantly correlated with coexisting infectious spondylitis and septic arthritis, especially in the patients displaying lower than 3.4 g/dL. Hypoalbuminemia could be simply a result of the deteriorated infection but also might be the cause of immune system dysfunction in the patients of our study group.

C-reactive protein, a well-known marker for detecting both local and systemic inflammation and infection, has been frequently used in daily clinical practice. CRP is helpful in detecting serious bacterial infections of fever of unknown origin and has been investigated as a predictive risk factor in infectious diseases, with differing outcomes and study populations reflecting all the different results. In orthopedics, acute infection, particularly sepsis, could be detected early and treated based on the CRP level checked during hospital admission. According to the study by Horino et al. [27], the persistently high CRP level was a predictive factor for hematogenous infections in univariant analysis. A similar result was also described by Lesen et al. [28], showing that sustained bacteremia was significantly related to metastatic infection, while the CRP level of the patients was usually higher than 100 mg/L. From the cohort study, Botheras et al. concluded that a CRP concentration of more than 161 mg/L on the day of admission was significantly associated with complications in community-associated *S. aureus* bacteremia [29]. The analytical results of our study revealed that a CRP level over 130 mg/L was significantly related with coexisting infections of infectious spondylitis and septic arthritis.

Prolonged antibiotics treatment before surgical intervention might indicate the delay in surgical timing after medical treatment failed. Although antibiotics therapy would avoid surgical-related complications, patients who need surgical debridement for infectious spondylitis might suffer from more complications and poor outcomes when long-term antibiotics treatment is not effective [30]. Secondary psoas muscle abscess and metastatic infection from hematogenous seeding are the most devastating complications from uncontrolled infectious spondylitis and septic arthritis. The present study revealed that patients with coexisting infections underwent a much longer duration of preoperative antibiotics treatment (22.1 ± 19.17 days) than the control group (7.26 ± 10.22 days). The statistical analysis showed that the risk of coexisting infections was largely increased after more than 8 days of preoperative antibiotics treatment in patients who needed surgical debridement. As the infection worsens, it might not only transmit through the bloodstream, but also directly spread into surrounding tissue. Clinically, psoas abscess is divided into two etiologies: primary psoas abscess, which is usually related to hematogenous seeding, and secondary psoas abscess, which is generally associated with contiguous spread, including gastrointestinal, genitourinary or even infectious spondylitis [1,31,32]. In our study, the incidence of positive psoas abscess was 80% in the study group, while in the control group it was only 14%. The statistical analysis revealed a *p*-value < 0.001, and we preferred psoas muscle abscess as a risk factor for coexisting infections. When the infectious spondylitis deteriorates, the infection foci might spread to the psoas muscle then further invade the hip joints. The iliopsoas bursa is located near the anterior capsule of the hip joint, which is one of the most vulnerable parts of the joint, and it is a potential route for the bacterium to spread and invade to the joint [33].

The prosthetic joint was one of the major risk factors for coexisting infections in the present study. The formation of biofilm on artificial prostheses is responsible for the predisposition of joint infections, especially in the patients with infectious spondylitis. Biofilm could provide the dense and defensive environment for living-in microorganisms to interact in different properties from blood-floating microorganisms of the same species [34]. Microorganisms from hematogenous seeding and adjacent tissue would enhance their colonization through interactions between microbial proteins and host proteins. Resistance to the host immune system and antimicrobial agents could be increased up to five thousand times in some cases [35]. From the study of Zimmerli et al., microorganisms were able to induce an infection with less than 100 colony-forming units under the protection of prosthetic biofilm [36]. This observation also certified our result that patients with total joint replacement had a greater risk of developing coexisting infections than the patients without a history of total joint replacement.

The present study is the first to investigate the risk factors of coexisting infectious spondylitis and septic arthritis, two of the most overwhelming infections in the orthopedic field. Five potential factors, including hypoalbuminemia (<3.4 g/dL), higher preoperative CRP (>130 mg/L), psoas muscle abscess, longer preoperative antibiotics treatment (>8 days) and history of total joint replacement, were figured out from the statistical analysis of the data of 351 patients over 8 years. Based on our results, patients with those five risk factors were potentially at a higher risk of being complicated with the coexisting infections spondylitis and septic arthritis. The current study does have some limitations which call for caution in the interpretation of its results. First, this study is a retrospective design due to the rarity of coexisting infectious spondylitis with septic arthritis, hence the small sample size of the study group. The uncommon occurrence of this disease precludes any other possible study designs, unless a multicenter effort is organized. Due to the retrospective nature of the study, bias and confounders are difficult to control and some essential patient data may not be available because data collection relies on retrospect and the written records, which led to the limited number of patients in the control group. In the future, a multicenter study can be performed to fully investigate predisposing risk factors of coexisting infectious spondylitis with septic arthritis to improve the patients’ outcomes and even prevent this rare condition.

## 5. Conclusions

Infectious spondylitis and septic arthritis are thought of as medical emergencies due to their high mortality and morbidity. Our study evaluated five risk factors as significant major findings: hypoalbuminemia (<3.4 g/dL), higher preoperative CRP (>130 mg/L), psoas muscle abscess, longer preoperative antibiotics treatment (>8 days) and history of total joint replacement. Clinicians should pay attention to the patients with these five factors to detect the coexisting infections as early as possible.

## Figures and Tables

**Table 1:** Demographic and pre-operative data of patients.

	Control Group (*n* = 50)	Study Group (*n* = 10)	*p*-Value	Odds Ratio
Age (years)	69.5 ± 8.11	63.06 ± 15.03	0.195	1.041
Sex (F/M)	30/20	6/4	1.000	1.000
Comorbidity				
DM	19	4	0.905	1.088
ESRD	4	0	0.355	0.920
Liver cirrhosis	2	2	0.064	6.000
Heart disease (CAD, heart failure, arrhythmia)	2	1	0.427	2.667
Rheumatological disease	1	0	0.652	0.980
Malignancy history	5	1	1.000	1.000
Total joint replacement history	2	4	<0.001 *	16.000
Preoperative data				
UTI (+)	8	4	0.083	3.500
Blood culture (+)	12	4	0.296	2.111
Pre-op anti(day)	7.26 ± 10.22	22.1 ± 19.17	0.038 *	1.072
Albumin (g/dL)	3.54 ± 0.73	2.87 ± 0.47	0.011 *	0.268
Creatinine	1.40 ± 1.84	0.98 ± 0.71	0.485	0.781
C-reactive protein (mg/L)	78.11 ± 82.14	184.89 ± 121.96	0.001 *	1.010
Platelet (1000/μL)	284.06 ± 133.62	380.5 ± 158.06	0.048 *	1.005
WBC (1000/μL)	10.52 ± 6.69	13.54 ± 3.02	0.169	1.066
Hemoglobin	11.17 ± 2.41	9.92 ± 1.75	0.223	0.793

The factors marked * refer to *p* < 0.05. Pre-op: pre-operative; UTI: urinary tract infection; DM: diabetes mellitus; ESRD: end-stage renal disease; WBC: white blood cell.

**Table 2:** Operation method and post-operative data.

	Control Group (*n* = 50)	Study Group (*n* = 10)	*p*-Value	Odds Ratio
Operation method				
Anterior	7	1	0.734	0.683
Posterior	40	8	1.000	1.000
Anterior + posterior	3	1	0.643	1.741
Post-operative, one month				
C-reactive protein (mg/L)	24.79 ± 35.75	29.57 ± 36.62	0.703	1.003
WBC	6.73 ± 1.93	7.64 ± 2.14	0.186	1.257
Platelet	267.57 ± 94.05	297.00 ± 95.82	0.406	1.003
Hemoglobin	11.51 ±1.36	12.08 ±1.15	0.223	1.369

WBC: white blood cell.

**Table 3:** Pre-operative image data.

	Control Group (*n* = 50)	Study Group (*n* = 10)	*p*-Value	Odds Ratio
Infection level			0.538	
Cervical	1	0		
Thoracic	8	3		
Lumbar	41	7		
Multiple level (≥2)	7	2	0.628	1.536
Psoas muscle abscess	7	8	<0.001 *	24.571
Epidural abscess	18	7	0.077	4.148

The factor marked * refers to *p* < 0.05.

**Table 4:** Culture data.

Pathogen	Control Group (*n* = 50)	Study Group (*n* = 10)	*p*-Value
*Staphylococcus aureus*	11	5	0.068
*Streptococcus species*	8	1	0.628
*E. coli*	4	0	
*Ps. Aeruginosa*	2	0	
*Kleb. Pneumoniae*	2	1	
*Enterococcus species*	2	1	
*Parvimonas micra*	3	0	
*Kodamaea ohmeri*	1	0	
NTM or TB	3	0	
No growth	14	2	

*E. coli*, *Escherichia coli*; *Ps. Aeruginosa*, *Pseudomonas aeruginosa*; *Kleb. Pneumoniae*, *Klebsiella pneumoniae*; NTM, Nontuberculous mycobacterium; TB, Tuberculosis.

## Data Availability

The datasets generated and/or analyzed during the current study are available from the corresponding author upon reasonable request.
